# Supplementary figures and images for: Convergent activation of the integrated stress response and ER–mitochondria uncoupling in VAPB-associated ALS
Source: EMBO Mol Med. 2025 Aug 5;17(9):2299–331. doi: 10.1038/s44321-025-00279-3 (PMC12423299; doi:10.1038/s44321-025-00279-3)

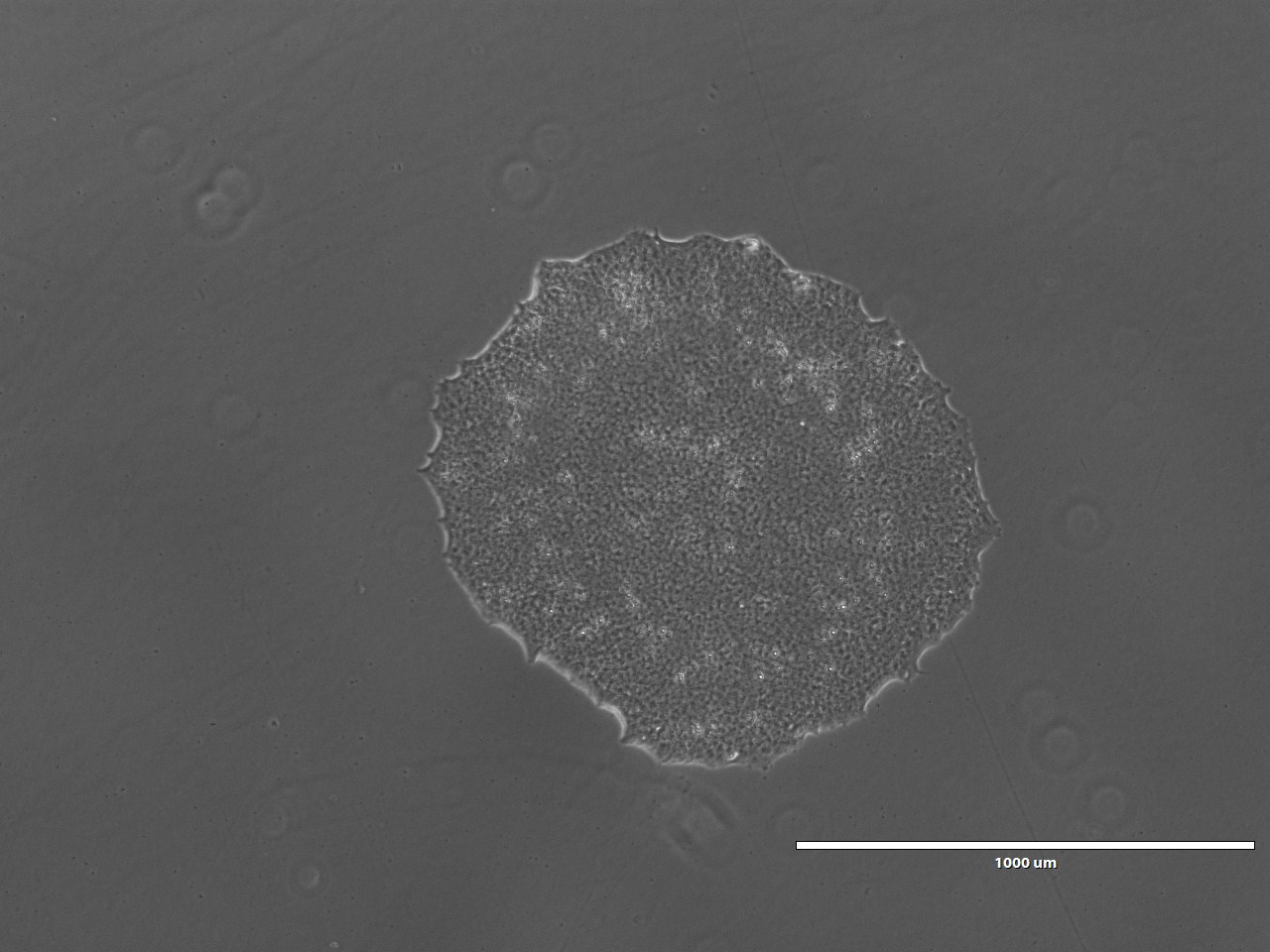

Supplement: Supplementary file 2 — Source data Fig. 1 [file 44321_2025_279_MOESM2_ESM.zip › Figure 1/1A/microscopy iPSC.tiff]

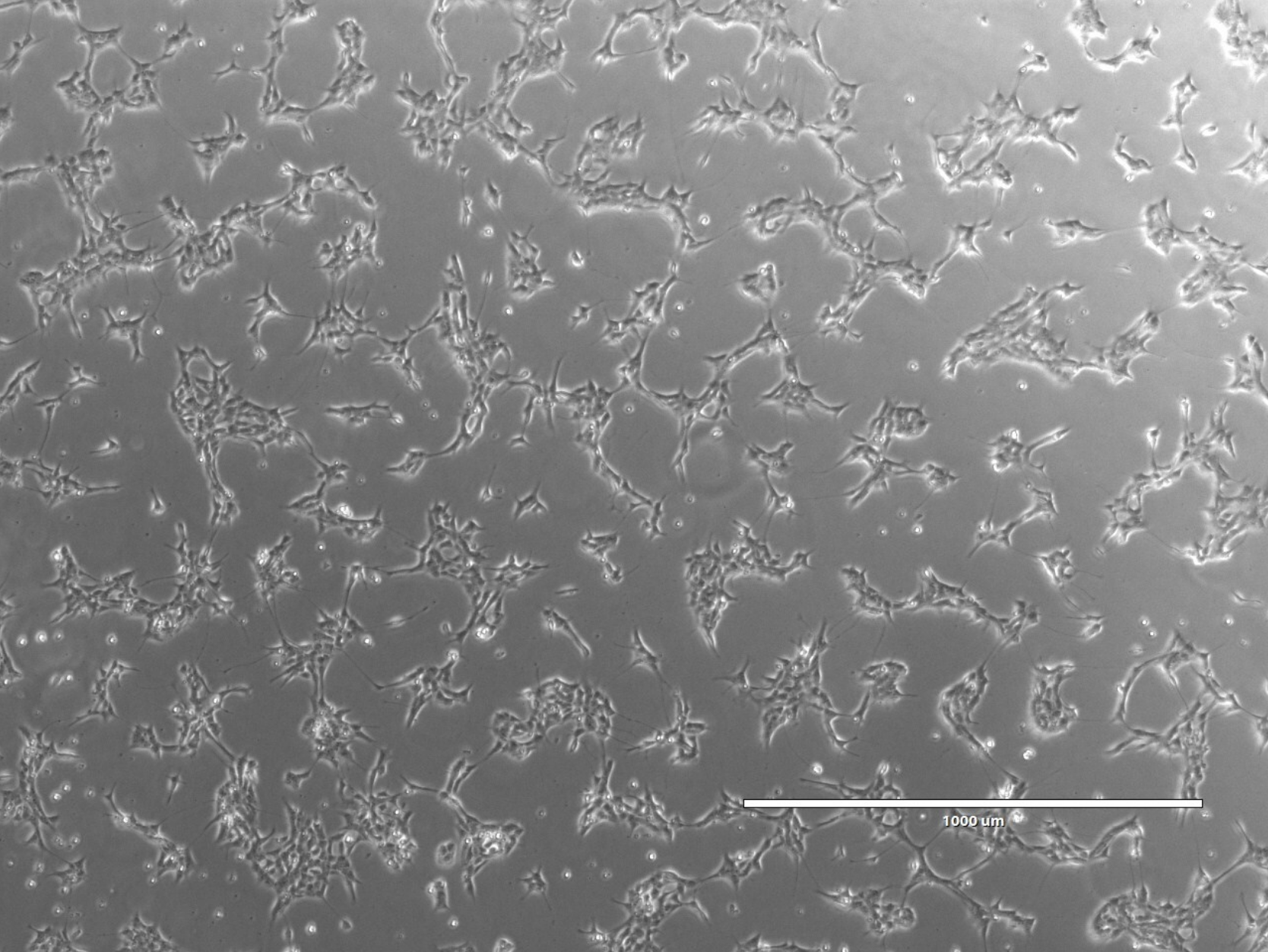

Supplement: Supplementary file 2 — Source data Fig. 1 [file 44321_2025_279_MOESM2_ESM.zip › Figure 1/1A/microscopy D16 MNP.tiff]

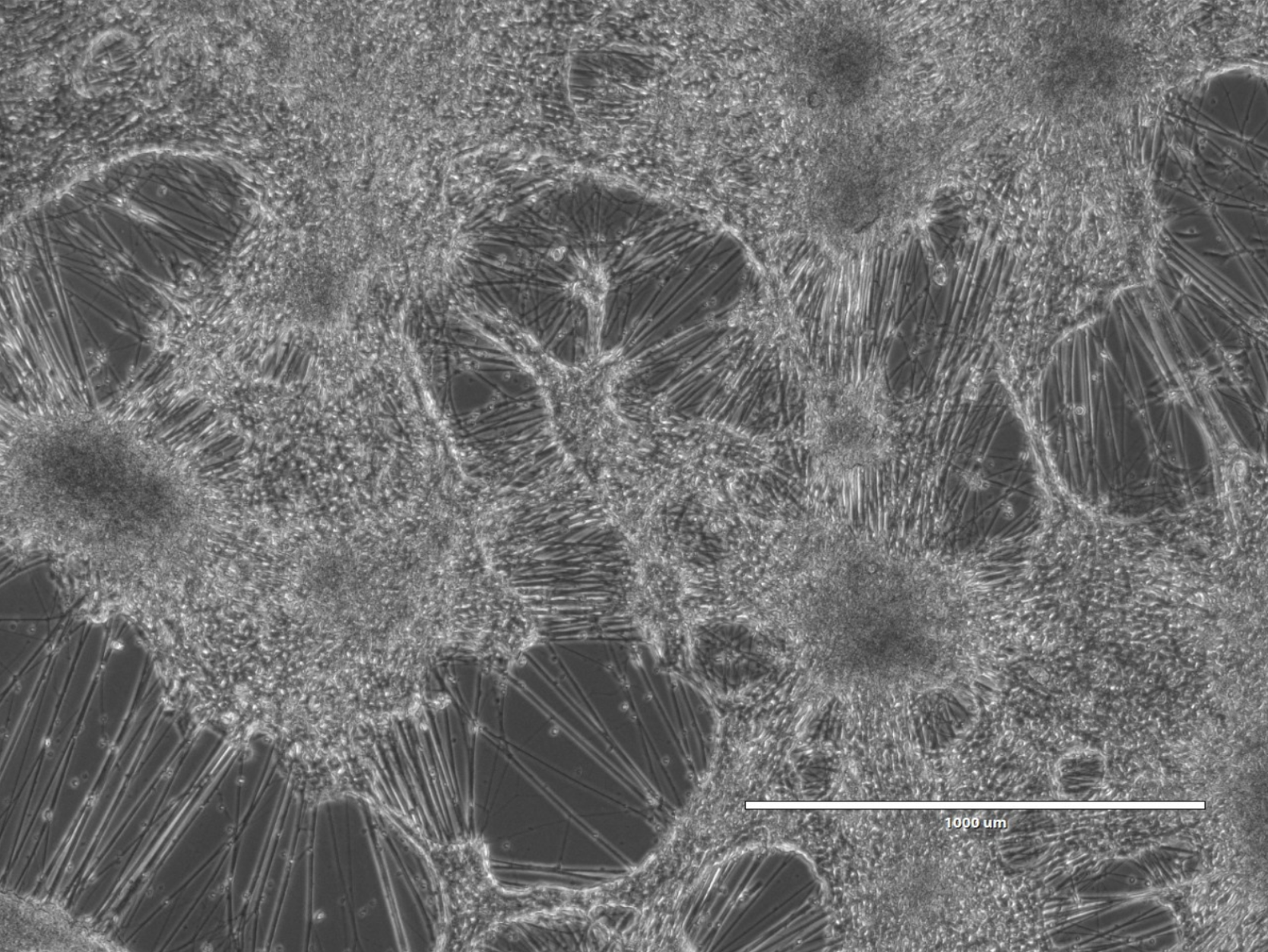

Supplement: Supplementary file 2 — Source data Fig. 1 [file 44321_2025_279_MOESM2_ESM.zip › Figure 1/1A/microscopy D30 MN.tiff]

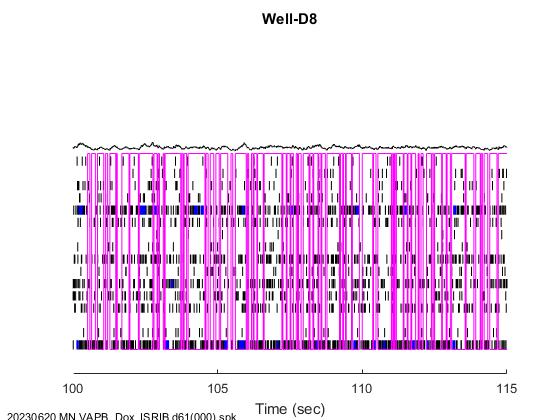

Supplement: Supplementary file 2 — Source data Fig. 1 [file 44321_2025_279_MOESM2_ESM.zip › Figure 1/1D/raster plot VAPB WT.tiff]

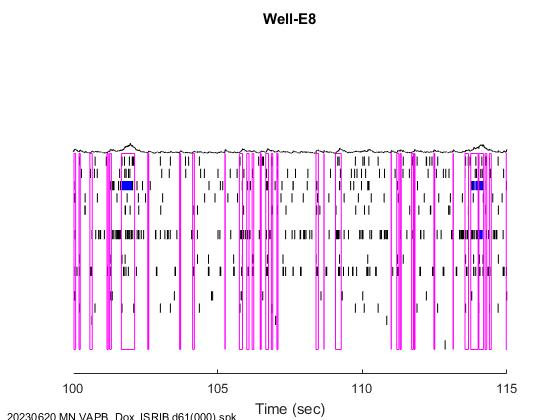

Supplement: Supplementary file 2 — Source data Fig. 1 [file 44321_2025_279_MOESM2_ESM.zip › Figure 1/1D/raster plot VAPB P56S.tiff]

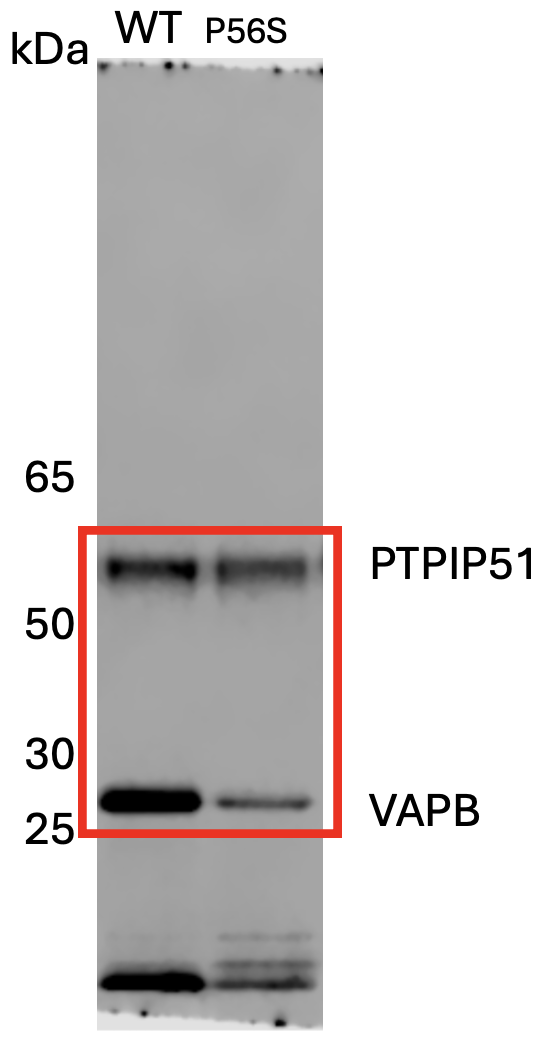

Supplement: Supplementary file 3 — Source data Fig. 2 [file 44321_2025_279_MOESM3_ESM.zip › Figure 2/2E/western PTPIP51 and VAPB.tiff]

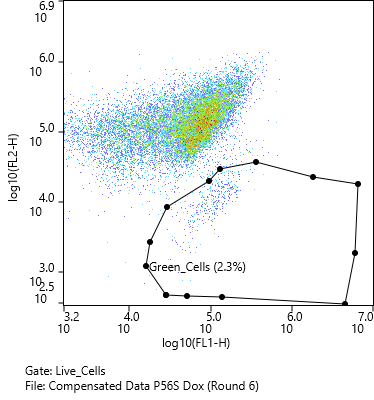

Supplement: Supplementary file 4 — Source data Fig. 3 [file 44321_2025_279_MOESM4_ESM.zip › Figure 3/3C/flow cytometry D30 VAPB P56S JC-1.tiff]

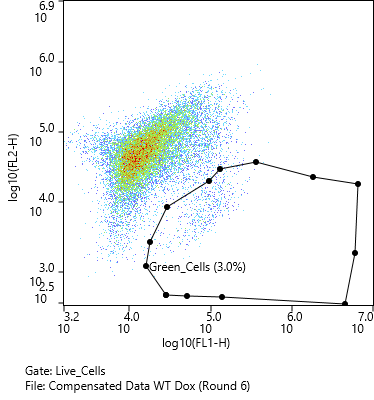

Supplement: Supplementary file 4 — Source data Fig. 3 [file 44321_2025_279_MOESM4_ESM.zip › Figure 3/3C/flow cytometry D30 VAPB WT JC-1.tiff]

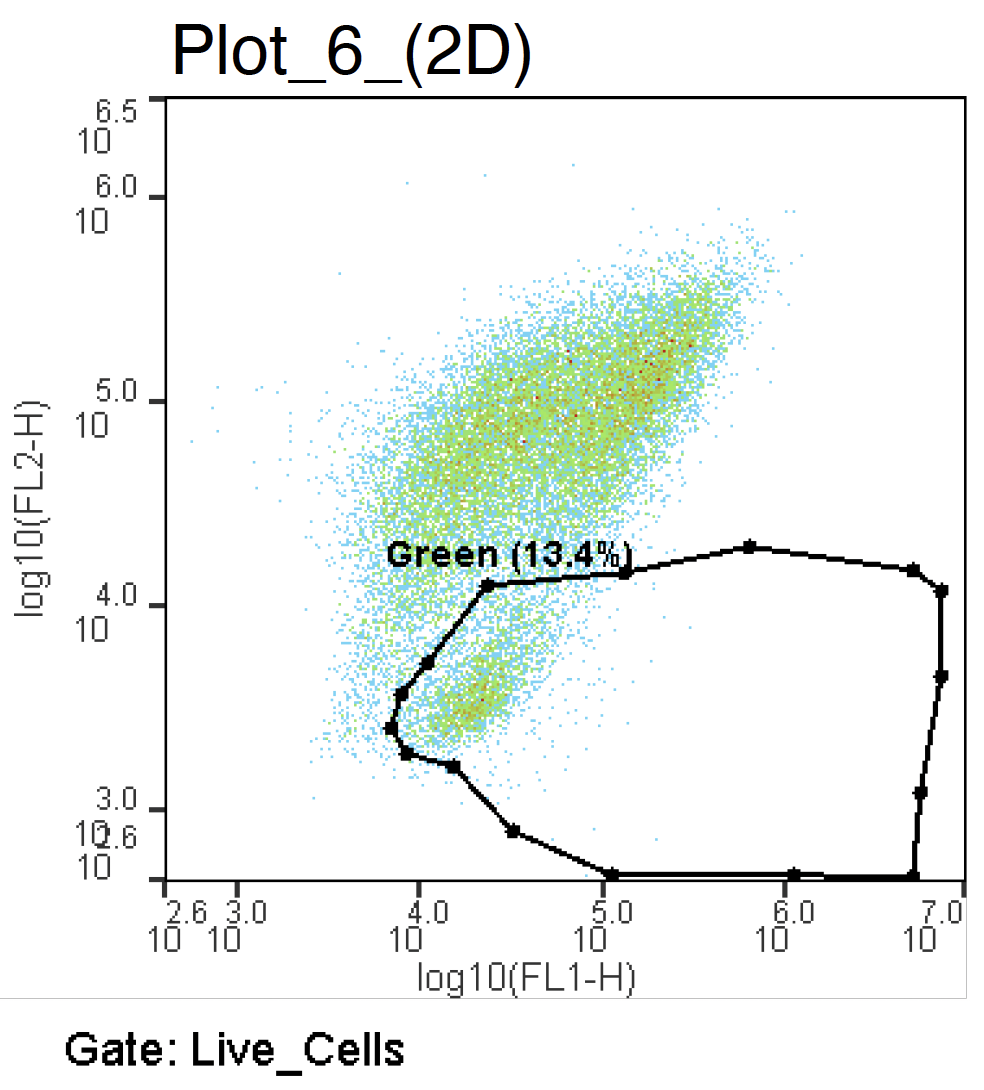

Supplement: Supplementary file 4 — Source data Fig. 3 [file 44321_2025_279_MOESM4_ESM.zip › Figure 3/3D/flow cytometry D60 VAPB P56S JC-1.tiff]

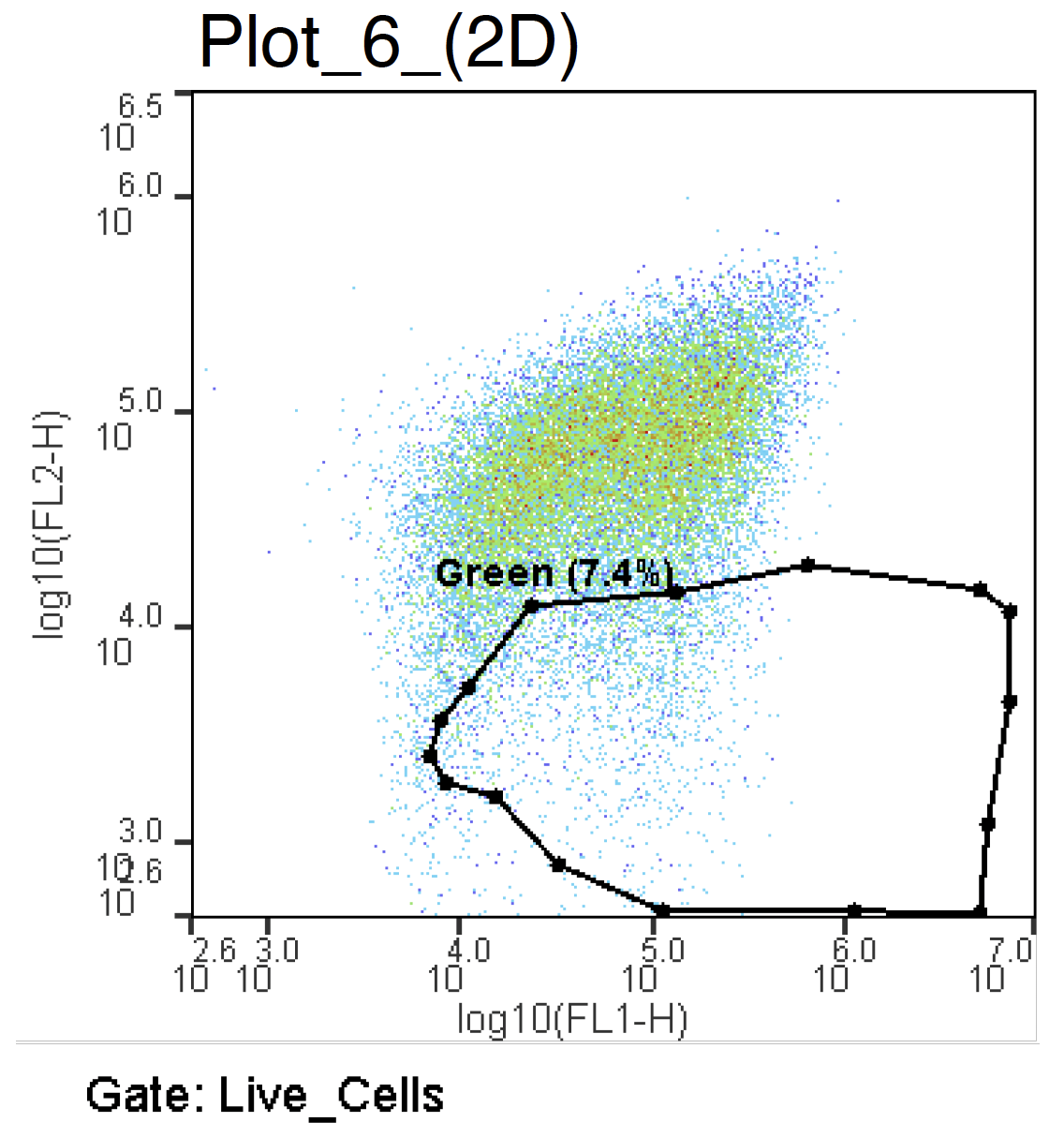

Supplement: Supplementary file 4 — Source data Fig. 3 [file 44321_2025_279_MOESM4_ESM.zip › Figure 3/3D/flow cytometry D60 VAPB WT JC-1.tiff]

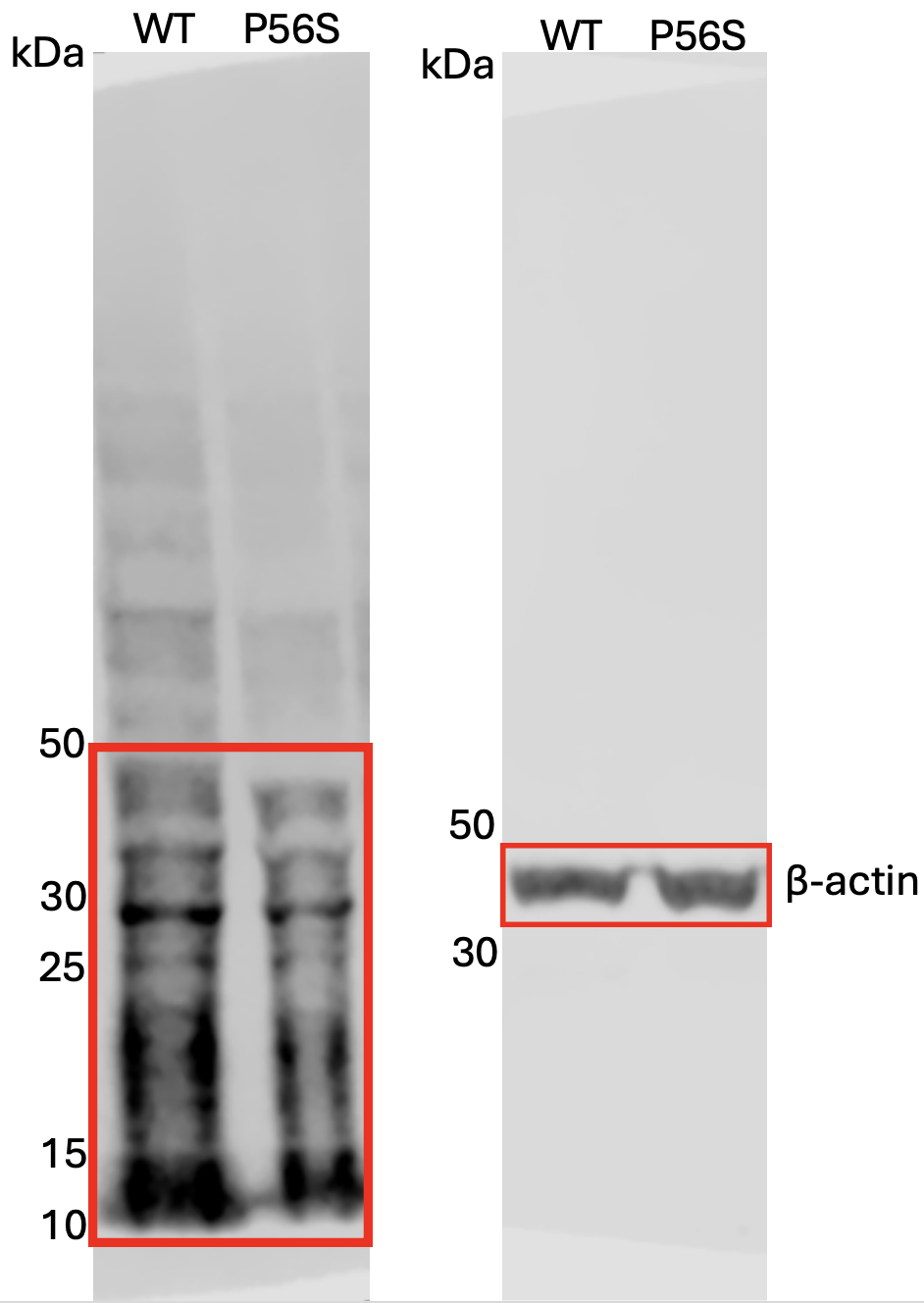

Supplement: Supplementary file 5 — Source data Fig. 4 [file 44321_2025_279_MOESM5_ESM.zip › Figure 4/4E/western SUnSET bactin.tiff]

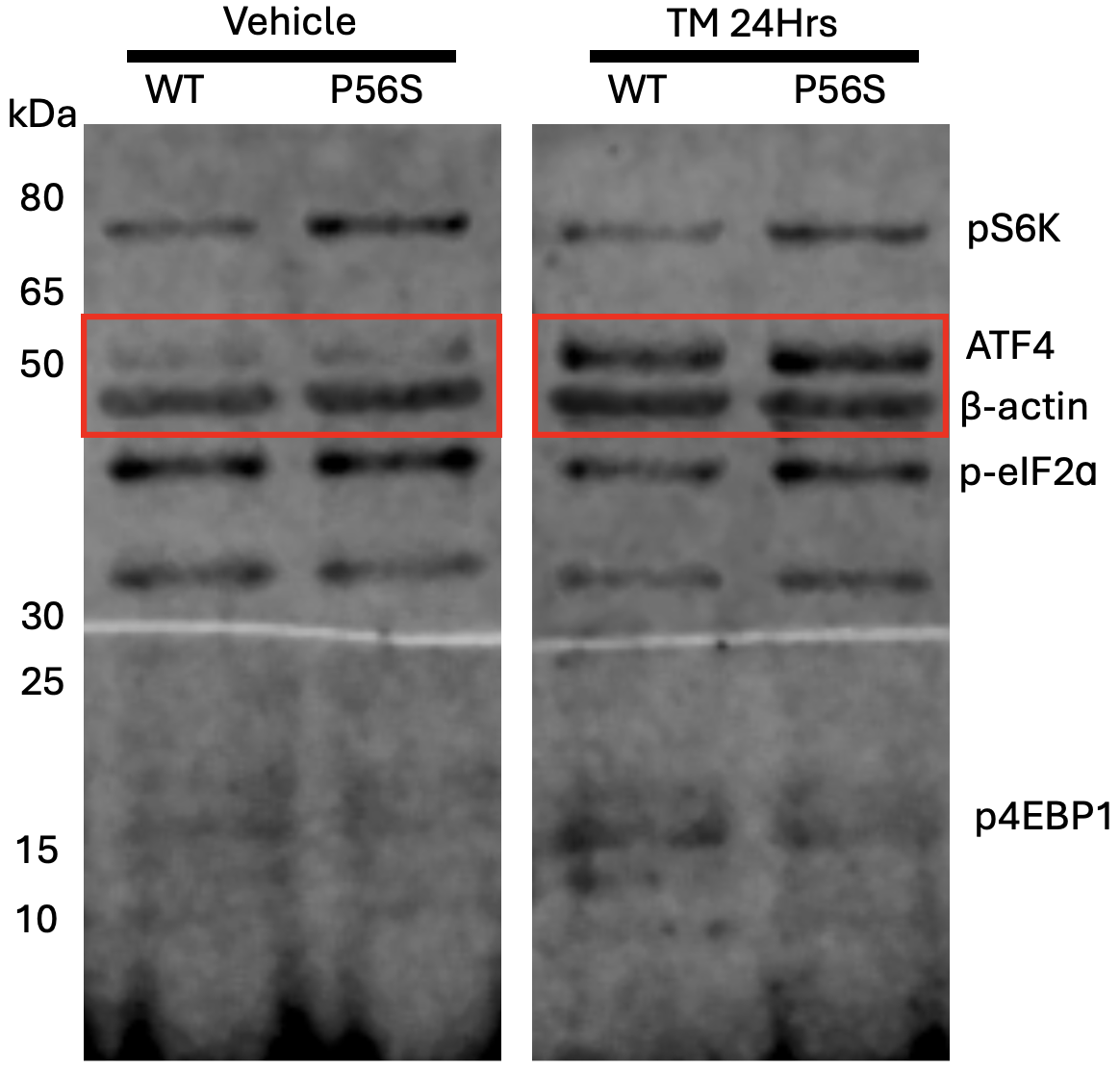

Supplement: Supplementary file 5 — Source data Fig. 4 [file 44321_2025_279_MOESM5_ESM.zip › Figure 4/4B/western ATF4 bactin.tiff]

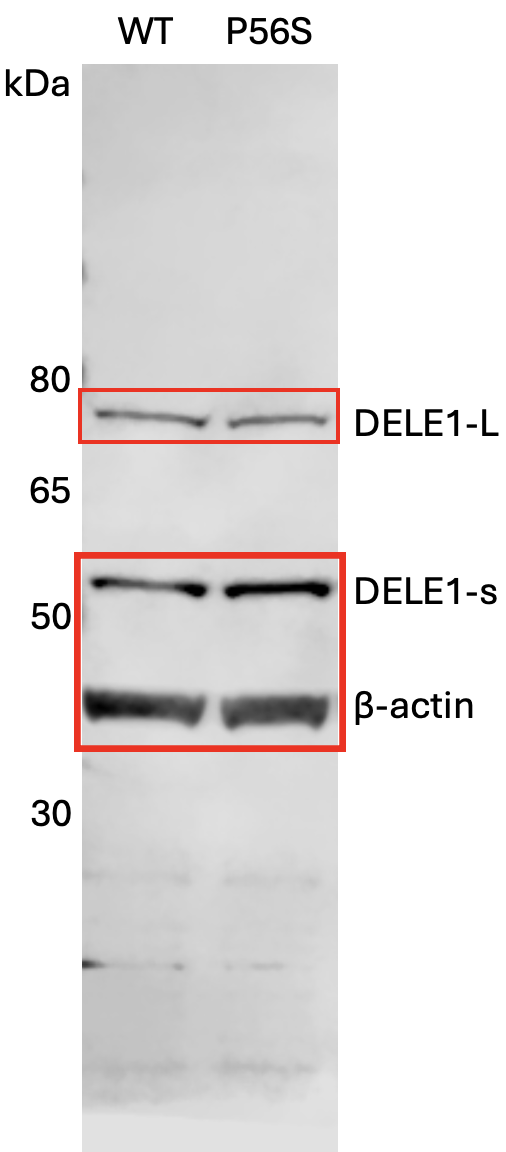

Supplement: Supplementary file 5 — Source data Fig. 4 [file 44321_2025_279_MOESM5_ESM.zip › Figure 4/4C/western DELE1-L DELE1-s.tiff]

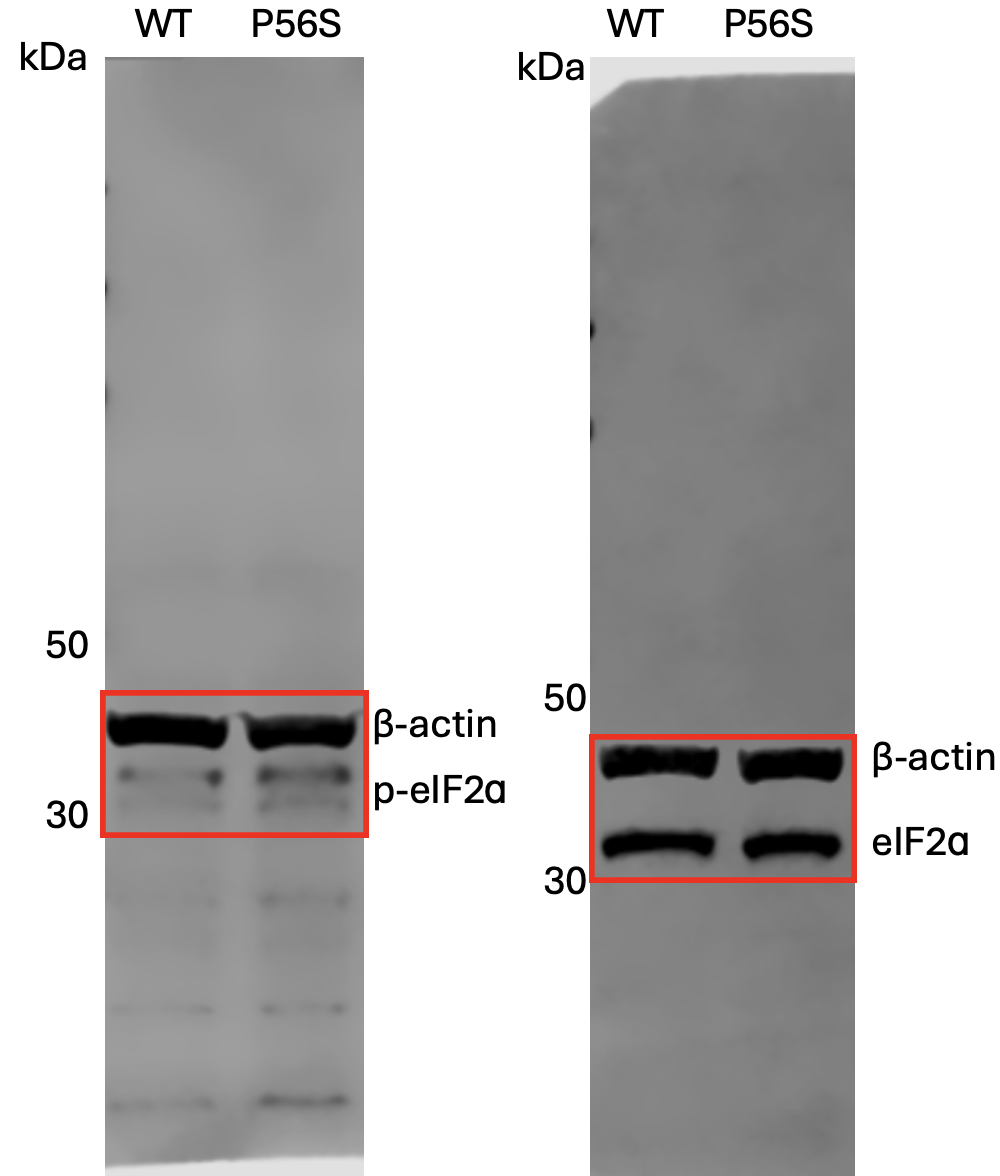

Supplement: Supplementary file 5 — Source data Fig. 4 [file 44321_2025_279_MOESM5_ESM.zip › Figure 4/4D/western p-eIF2a eIF2a.tiff]

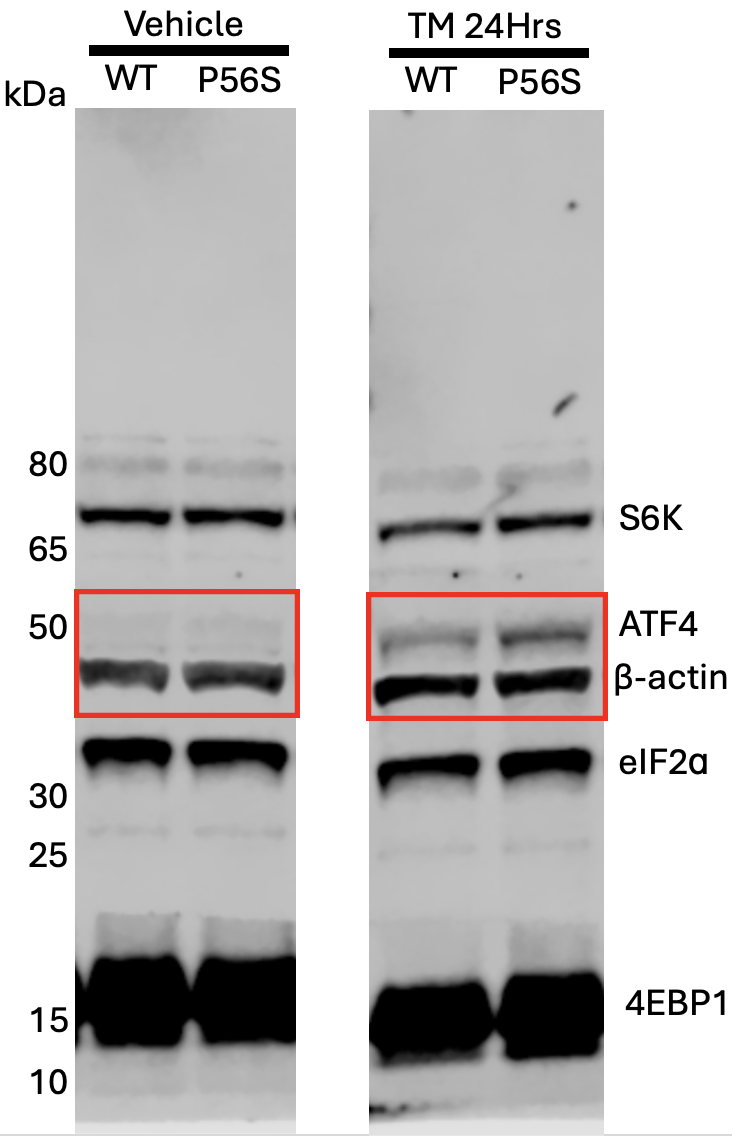

Supplement: Supplementary file 5 — Source data Fig. 4 [file 44321_2025_279_MOESM5_ESM.zip › Figure 4/4A/western ATF4 bactin.tiff]

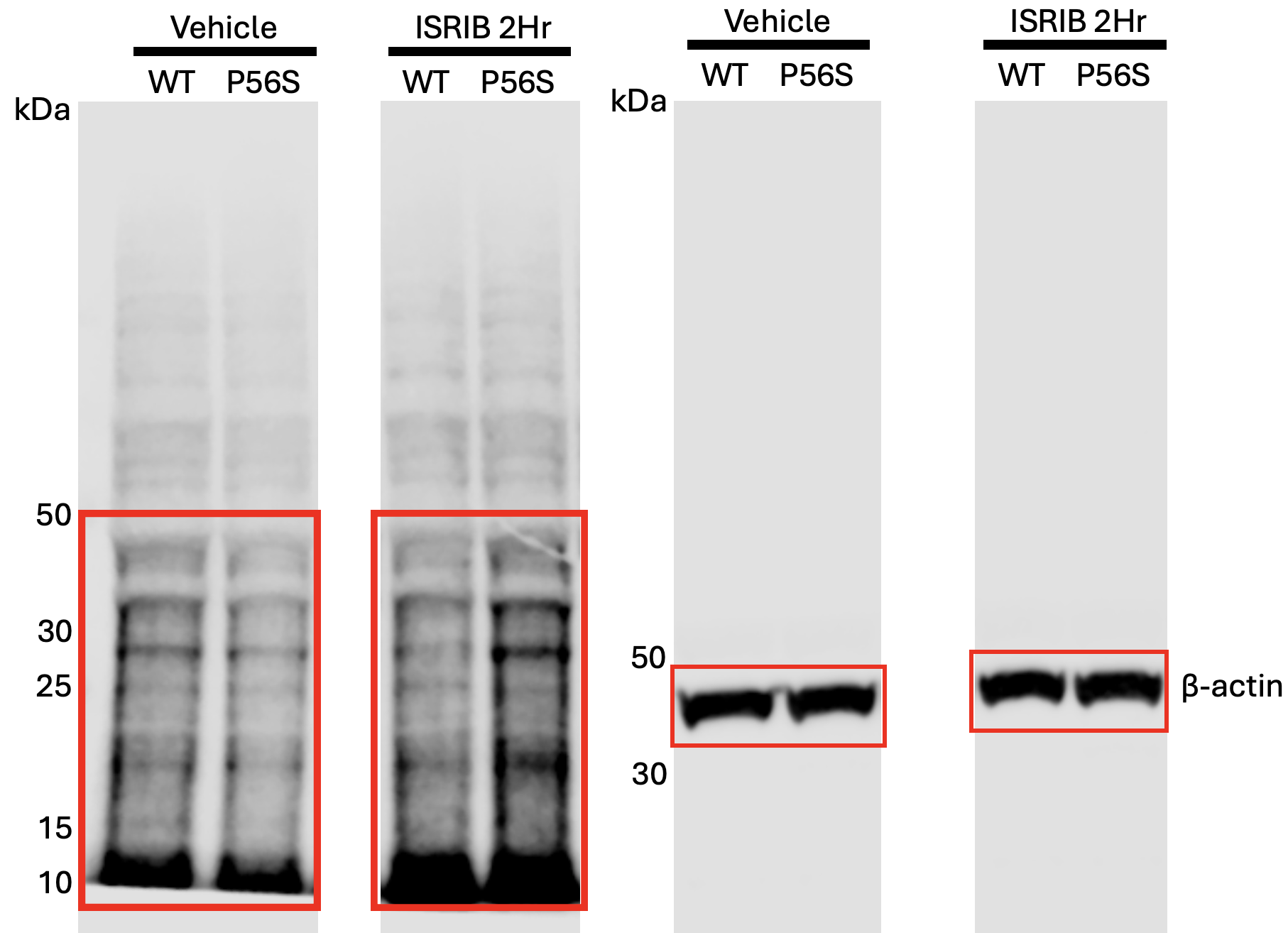

Supplement: Supplementary file 6 — Source data Fig. 5 [file 44321_2025_279_MOESM6_ESM.zip › Figure 5/5A/western SUnSET Bactin.tiff]

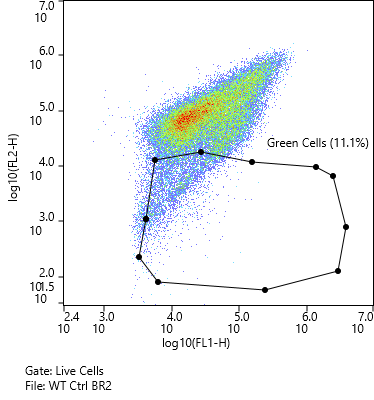

Supplement: Supplementary file 6 — Source data Fig. 5 [file 44321_2025_279_MOESM6_ESM.zip › Figure 5/5B/flow cytometry D60 VAPB WT Vehicle JC-1.tiff]

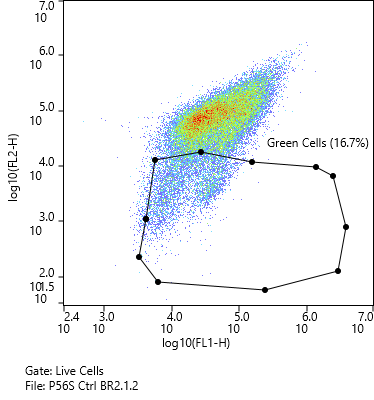

Supplement: Supplementary file 6 — Source data Fig. 5 [file 44321_2025_279_MOESM6_ESM.zip › Figure 5/5B/flow cytometry D60 VAPB P56S Vehicle JC-1.tiff]

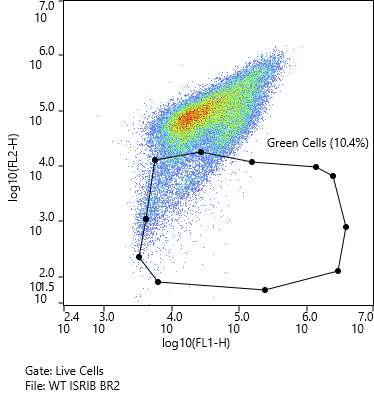

Supplement: Supplementary file 6 — Source data Fig. 5 [file 44321_2025_279_MOESM6_ESM.zip › Figure 5/5B/flow cytometry D60 VAPB WT ISRIB JC-1.tiff]

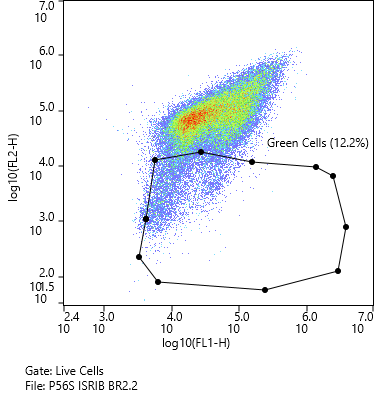

Supplement: Supplementary file 6 — Source data Fig. 5 [file 44321_2025_279_MOESM6_ESM.zip › Figure 5/5B/flow cytometry D60 VAPB P56S ISRIB JC-1.tiff]

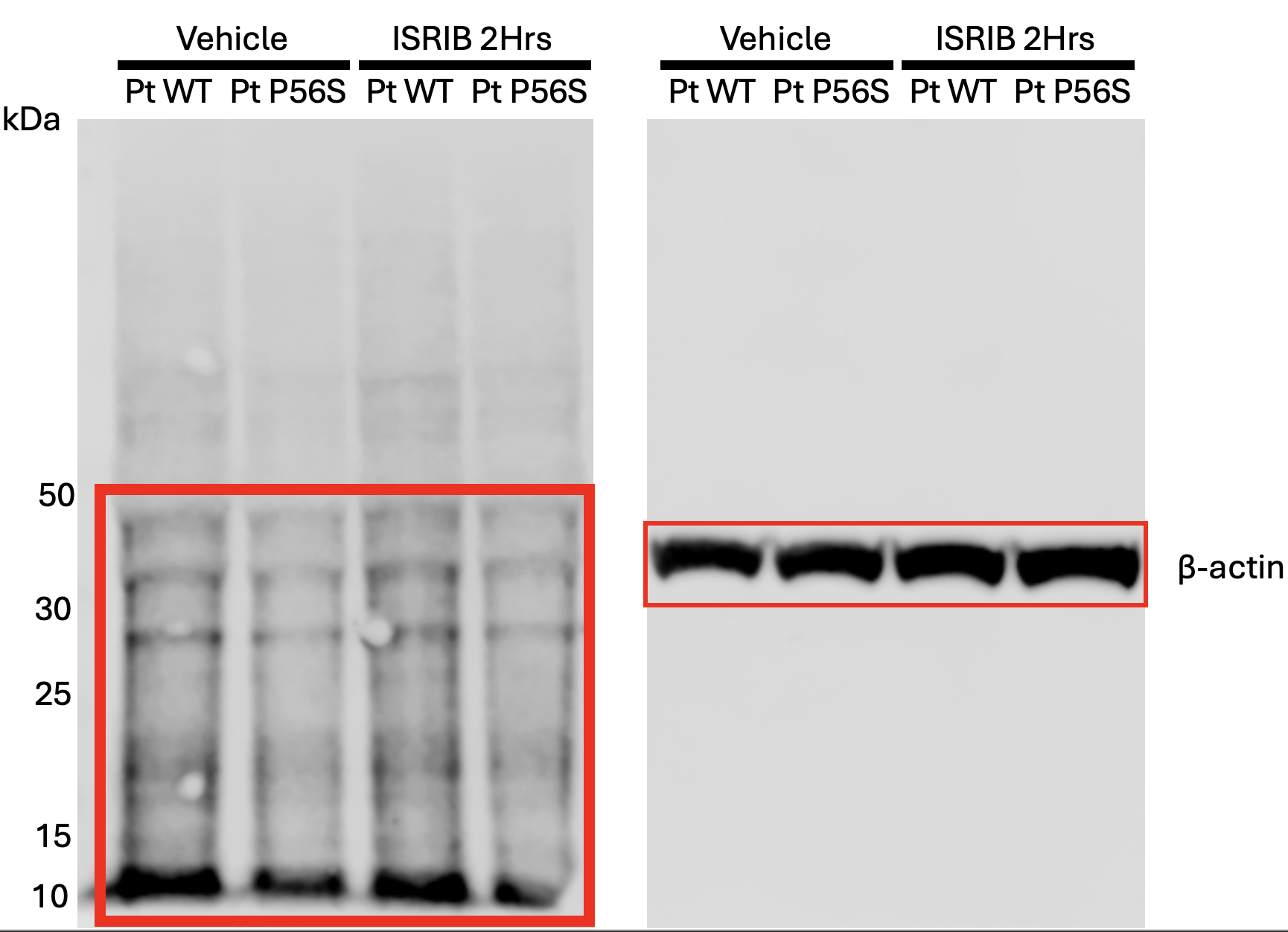

Supplement: Supplementary file 7 — Source data Fig. 6 [file 44321_2025_279_MOESM7_ESM.zip › Figure 6/6A/western SUnSET bactin.tiff]

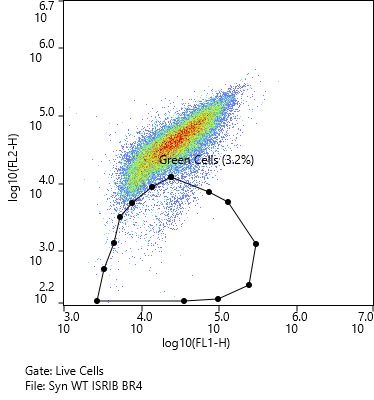

Supplement: Supplementary file 7 — Source data Fig. 6 [file 44321_2025_279_MOESM7_ESM.zip › Figure 6/6B/flow cytometry D60 Pt VAPB WT ISRIB JC-1.tiff]

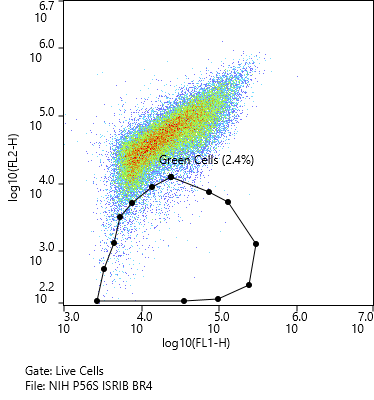

Supplement: Supplementary file 7 — Source data Fig. 6 [file 44321_2025_279_MOESM7_ESM.zip › Figure 6/6B/flow cytometry D60 Pt VAPB P56S ISRIB JC-1.tiff]

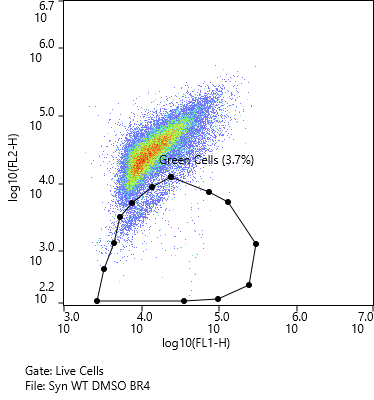

Supplement: Supplementary file 7 — Source data Fig. 6 [file 44321_2025_279_MOESM7_ESM.zip › Figure 6/6B/flow cytometry D60 Pt VAPB WT Vehicle JC-1.tiff]

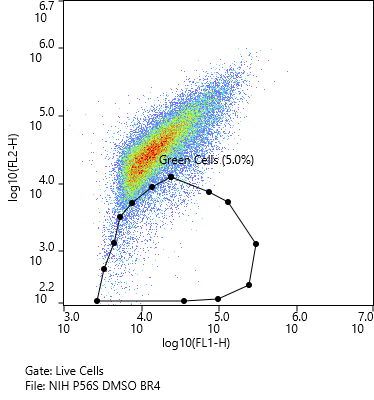

Supplement: Supplementary file 7 — Source data Fig. 6 [file 44321_2025_279_MOESM7_ESM.zip › Figure 6/6B/flow cytometry D60 Pt VAPB P56S Vehicle JC-1.tiff]
