# Supplementary material for: Convergent activation of the integrated stress response and ER–mitochondria uncoupling in VAPB-associated ALS
Source: EMBO Mol Med. 2025 Aug 5;17(9):2299–331. doi: 10.1038/s44321-025-00279-3 (PMC12423299; doi:10.1038/s44321-025-00279-3)
Supplement: Supplementary file 9 — Expanded View Figures [file 44321_2025_279_MOESM9_ESM.pdf]

Expanded View Figures

Figure EV1. Characterization of doxycycline-inducible VAPB iPSCs.

(**A**) Next-generation sequencing of patient iPSCs following CRISPR-Cas9-mediated mutagenesis revealed two frameshift mutations: a 2 bp deletion in the WT VAPB allele and a 1 bp insertion in the P56S allele. (**B**) Sanger sequencing of VAPB WT and VAPB P56S lines after transduction with doxycycline-inducible constructs; the P56S mutation is highlighted in red. (**C**) Immunofluorescence analysis confirming robust expression of pluripotency markers Nanog, SOX2, and Oct4 in all inducible lines. $N = 3$ biological replicates, minimum of three technical replicates. (**D**) Quantitative PCR of HA-tagged VAPB transcripts in doxycycline-inducible iPSC lines. Expression was normalized to Neomycin and RPL3. Data were shown as mean ± SEM; $N = 3$ qPCR biological replicates per line, per time point.

 © The Author(s)

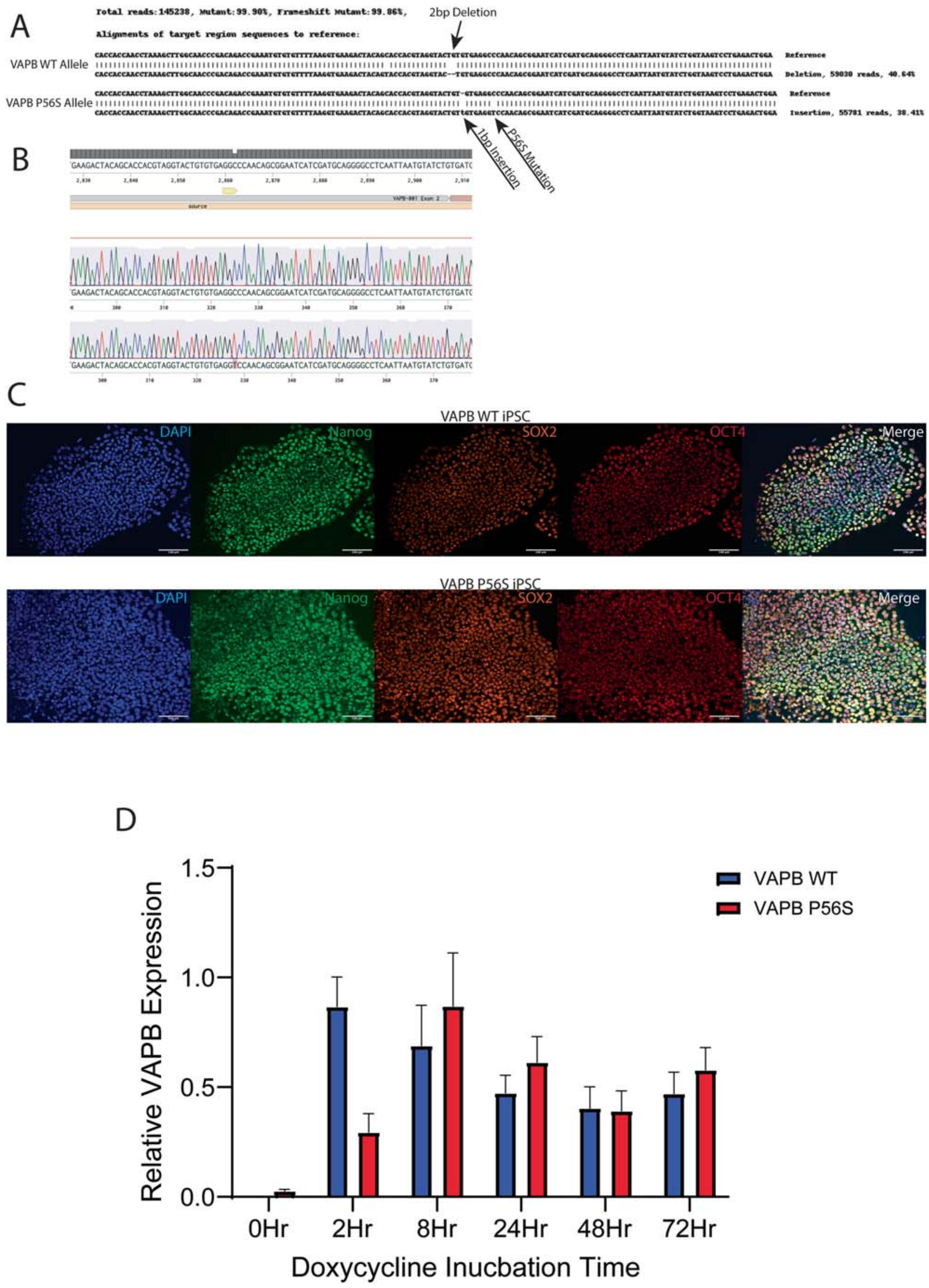
A
Total reads:145238, Mutant:99.90%, Frameshift Mutant:99.86%,
Alignments of target region sequences to reference:
2bp Deletion
VAPB WT Allele
Reference
Deletion, 59030 reads, 40.64%
VAPB P56S Allele
Reference
Insertion, 55781 reads, 38.41%
1bp Insertion
P56S Mutation
B
source
C
VAPB WT iPSC
DAPI
Nanog
SOX2
OCT4
Merge
VAPB P56S iPSC
DAPI
Nanog
SOX2
OCT4
Merge
D
VAPB WT
VAPB P56S
Relative VAPB Expression
1.5
1.0
0.5
0.0
0Hr
2Hr
8Hr
24Hr
48Hr
72Hr
Doxycycline Inucbation Time

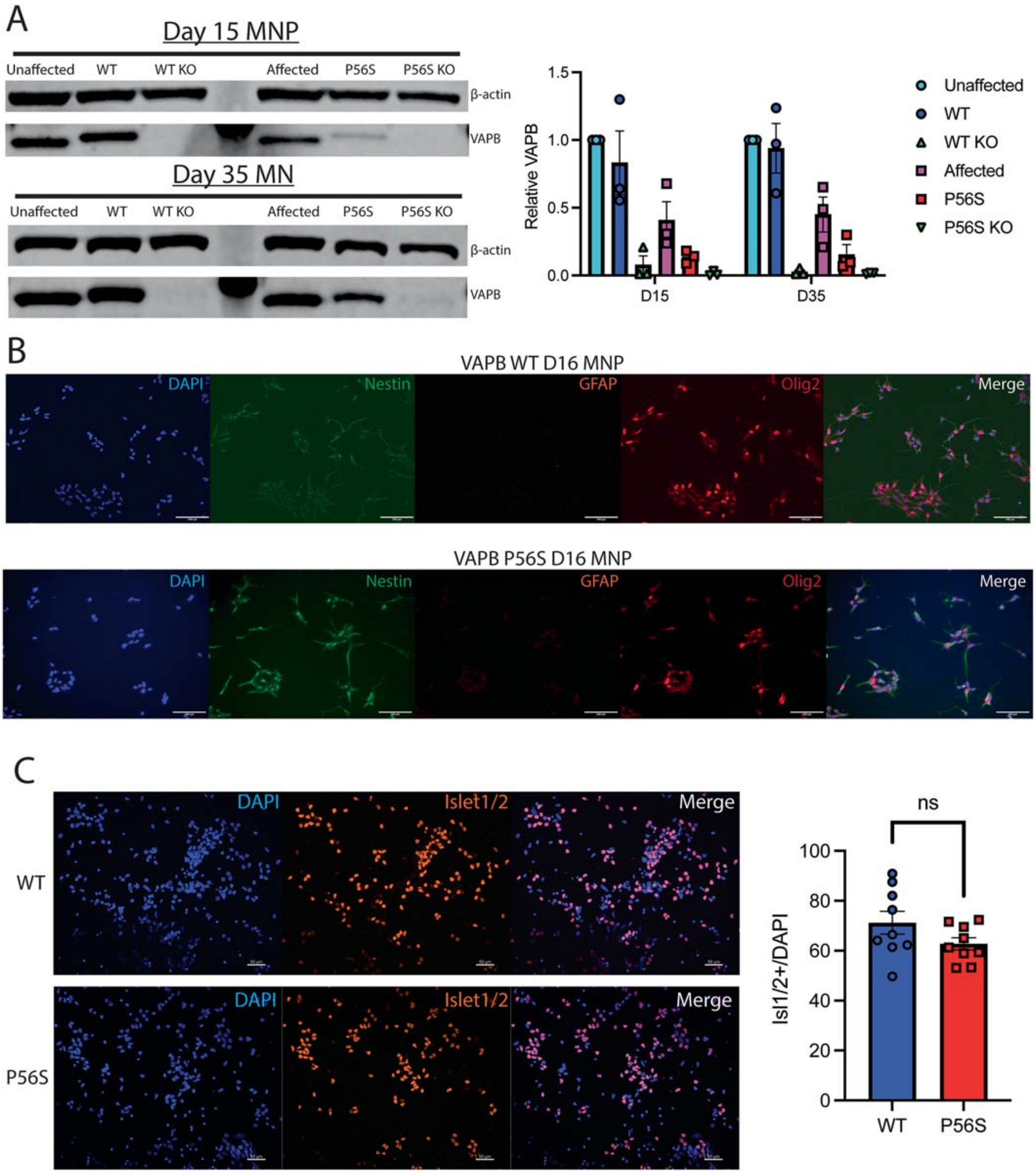

Figure EV2. Characterization of doxycycline-inducible VAPB iPSC-derived motor neurons.

(**A**) Western blot analysis of VAPB expression at days 15 (motor neuron progenitors, MNP) and 30 of differentiation. "Unaffected" and "Affected" refer to familial patient iPSC lines, with inducible lines generated from the Affected line. Data were presented as mean ± SEM; $N = 3$ biological replicates. (**B**) Immunofluorescence on day 15 showing expression of motor neuron progenitor markers Nestin and Olig2, with no detectable expression of the glial marker GFAP. $N = 3$ biological replicates, minimum of three technical replicates. (**C**) Immunofluorescence on day 30 comparing the number of DAPI/Islet 1/2 double-positive cells. Statistical analysis: unpaired *t*-test, no significant differences were observed ($p > 0.05$). Data were shown as mean ± SEM; $N = 3$ biological replicates, minimum of three technical replicates.

 © The Author(s)

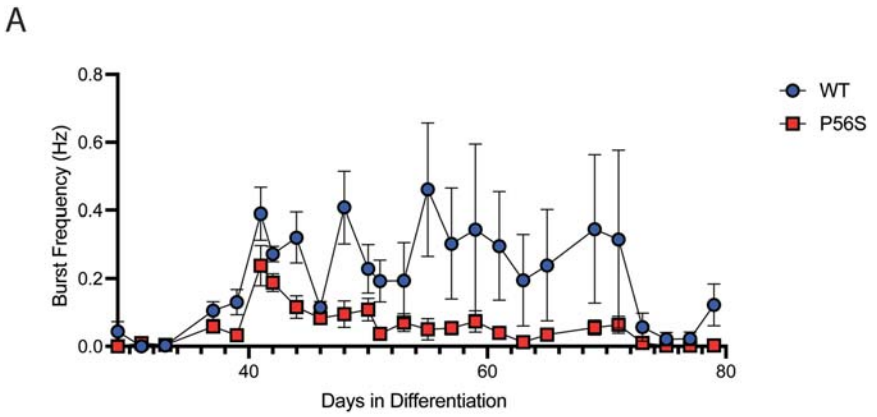

Figure EV3. Burst frequency of VAPB P56S iPSC-derived motor neurons across differentiation.

(**A**) Data presented as mean ± SEM; $N = 8$ technical replicates.

© The Author(s)

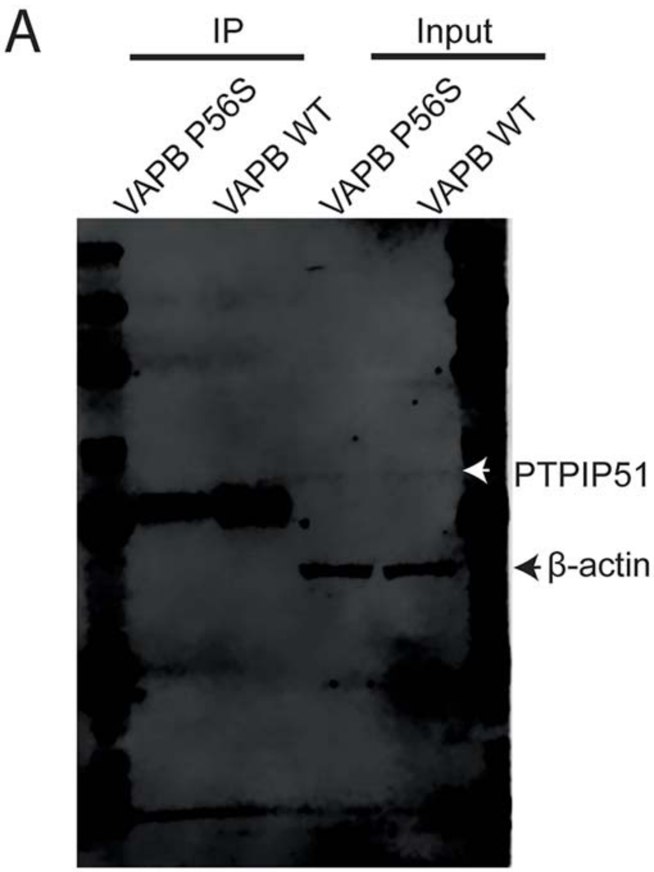

Figure EV4. Overexposed VAPB co-immunoprecipitation western blot.

(**A**) Overexposed western blot showing the PTPIP51 band in the input lane and absence of β-actin in the IP lane.

 © The Author(s)

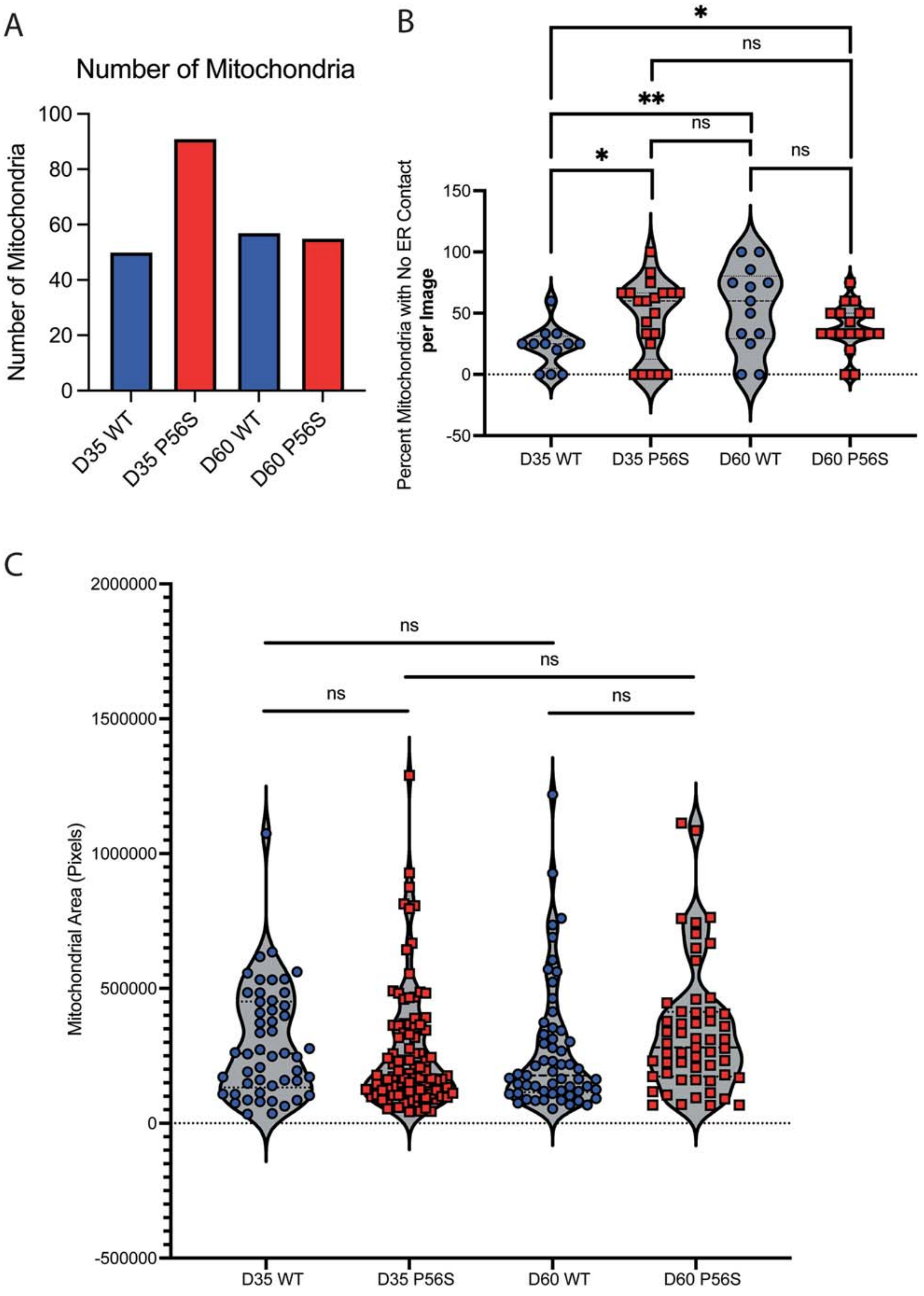
A
Number of Mitochondria
Number of Mitochondria
100
80
60
40
20
0
D35 WT
D35 P56S
D60 WT
D60 P56S
B
*
ns
**
ns
*
ns
Percent Mitochondria with No ER Contact per Image
150
100
50
0
-50
D35 WT
D35 P56S
D60 WT
D60 P56S
C
ns
ns
ns
ns
Mitochondrial Area (Pixels)
2000000
1500000
1000000
500000
0
-500000
D35 WT
D35 P56S
D60 WT
D60 P56S

Figure EV5. Expanded quantification of ER-mitochondria contacts (MAMs) from electron microscopy.

(**A**) Number of mitochondria counted for each line across all images taken at time point (day 35 or day 60) from 1 experiment. (**B**) Percent of mitochondria present in each image with no ER-Mitochondrial contact observable. Statistical analysis: unpaired *t*-test: VAPB WT Day 35 vs VAPB P56S Day 35 $p = 0.0248$*, VAPB WT Day 35 vs VAPB WT Day 60 $p = 0.0078$**, VAPB WT Day 35 vs VAPB P56S Day 60 $p = 0.0293$*, Violin plot with median, first and third quartiles denoted as dashed lines, $N = 37$ mitochondria from 13 unique images for D35 WT, 42 mitochondria from 21 unique images for D35 P56S, 24 mitochondria from 13 unique images for D60 WT, 31 mitochondria for 18 unique images for D60 P56S. (**C**) Area of mitochondria in pixels. Statistical analysis: unpaired *t*-test: all *p* values >0.05. Violin plot with median, first and third quartiles denoted as dashed lines, $N = 50$ mitochondria from 13 unique images for D35 WT, 91 mitochondria from 21 unique images for D35 P56S, 57 mitochondria from 13 unique images for D60 WT, 55 mitochondria from 18 unique images for D60 P56S.

© The Author(s)

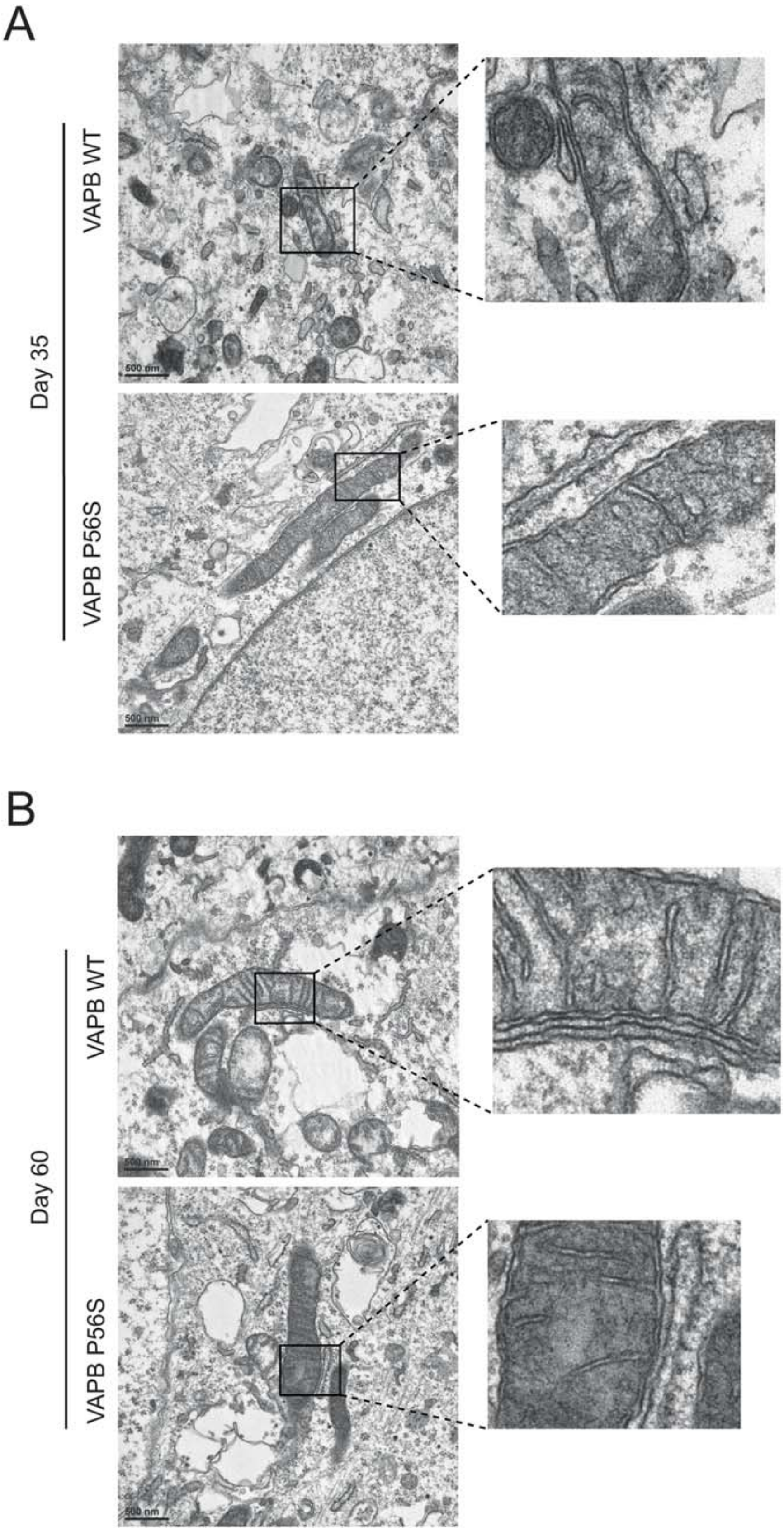
A
Day 35
VAPB WT
VAPB P56S
500 nm
500 nm
B
Day 60
VAPB WT
VAPB P56S
500 nm
500 nm

Figure EV6. Additional electron micrographs of ER-mitochondria contacts during motor neuron differentiation.

(**A**) Representative images from day 35 VAPB WT and VAPB P56S motor neurons, with insert zooms highlighting ER-mitochondria contacts. (**B**) Representative images from day 60 VAPB WT and VAPB P56S motor neurons, with insert zooms as above. Source data are available online for this figure.

© The Author(s)

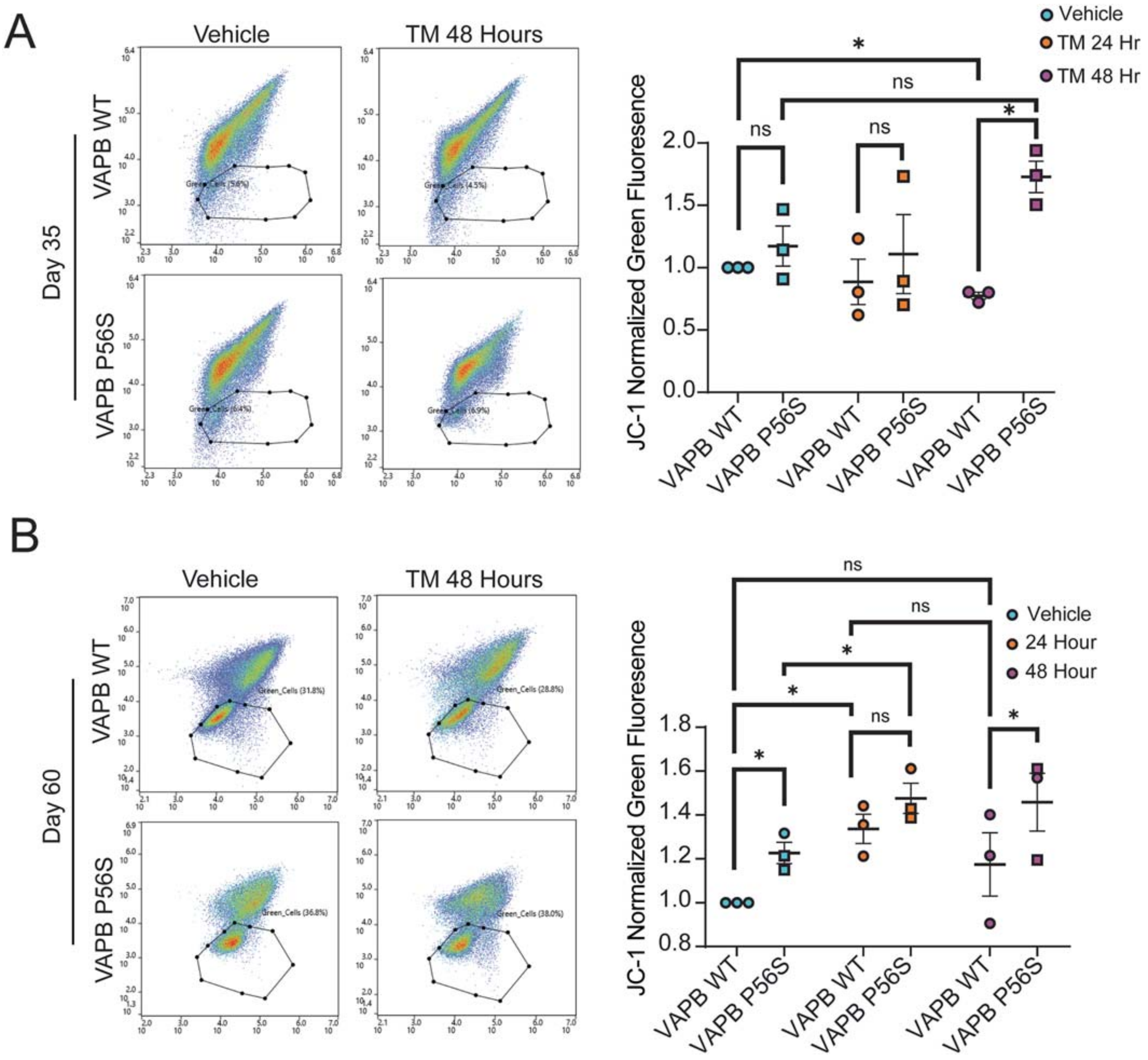

Figure EV7. VAPB P56S impairs cellular response to stress.

(**A**) JC-1 staining of day 35 motor neurons after 24 or 48 h of tunicamycin treatment, or untreated control. Data were presented as mean ± SEM; $N = 3$ biological replicates. Statistical analysis: paired *t*-test. Significant comparisons: VAPB WT Vehicle vs VAPB WT TM 48 h ($p = 0.0141$), VAPB WT TM 48 h vs VAPB P56S TM 48 h ($p = 0.0450$). (**B**) Same analysis as (**A**) on day 60. Statistical analysis: paired *t*-test. Significant comparisons: VAPB WT Vehicle vs VAPB P56S Vehicle ($p = 0.043$), VAPB WT Vehicle vs VAPB WT TM 24 h ($p = 0.0374$), VAPB P56S Vehicle vs VAPB P56S TM 24 h ($p = 0.0229$), VAPB WT TM 48 h vs VAPB P56S TM 48 h ($p = 0.0212$).

© The Author(s)

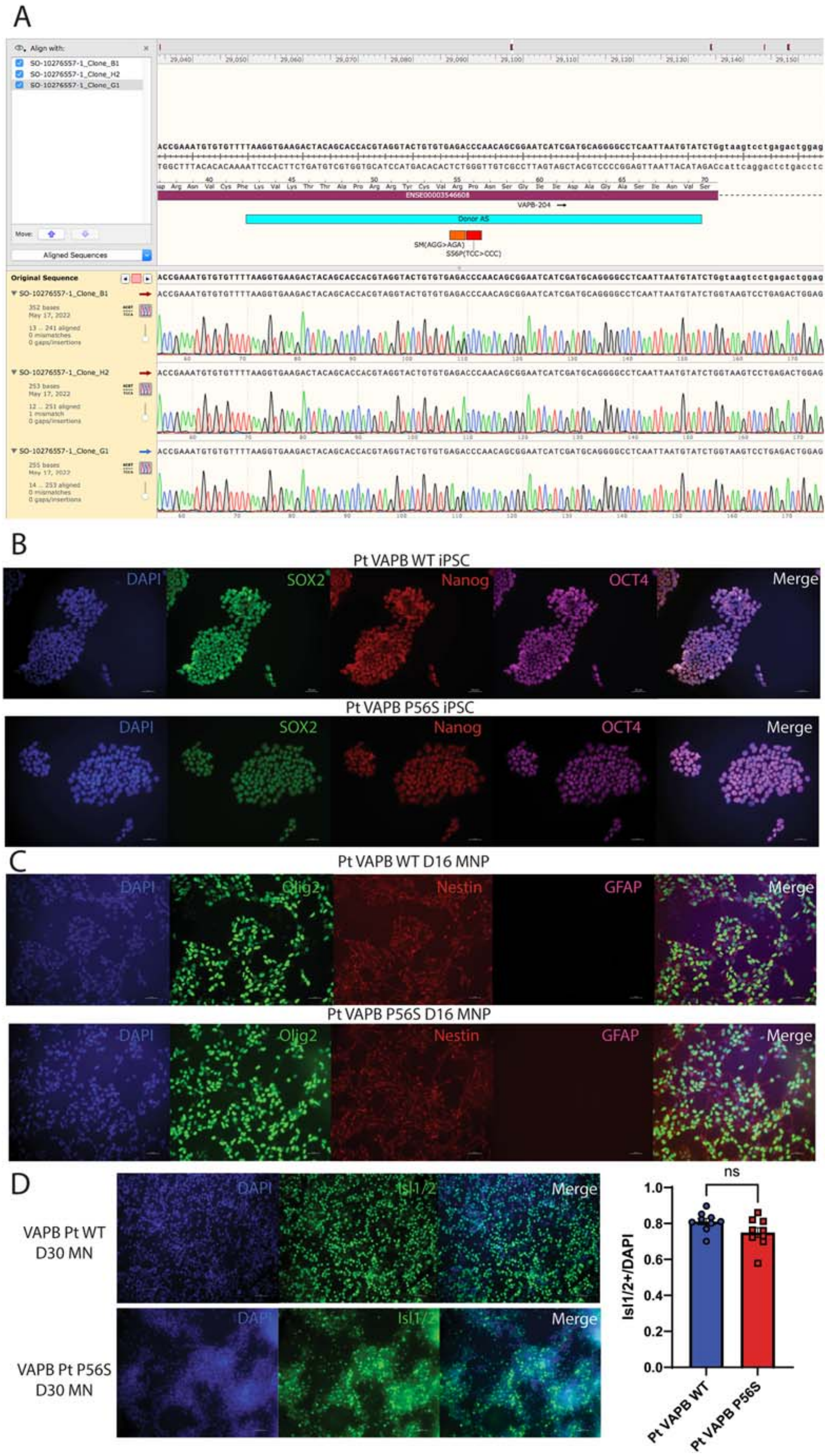
A
B
Pt VAPB WT iPSC
DAPI
SOX2
Nanog
OCT4
Merge
Pt VAPB P56S iPSC
C
Pt VAPB WT D16 MNP
Olig2
Nestin
GFAP
Pt VAPB P56S D16 MNP
D
VAPB Pt WT D30 MN
VAPB Pt P56S D30 MN
Isl1/2
ns
Isl1/2+/DAPI
Pt VAPB WT
Pt VAPB P56S

 © The Author(s)

Figure EV8. Characterization of VAPB patient iPSCs and isogenic control lines.

(**A**) Sanger sequencing of the patient iPSC line following CRISPR-Cas9-mediated mutagenesis. The heterozygous P56S mutation (TCC, encoding Ser) was corrected to the WT codon (CCC, encoding Pro). A synonymous mutation (SM) was also introduced at the adjacent PAM site (AGG to AGA, both encoding Arg) via the donor template to prevent re-cutting by Cas9. (**B**) Immunofluorescence confirming expression of pluripotency markers (SOX2, Nanog, and OCT4) in isogenic iPSC lines. $N = 3$ biological replicates, minimum of three technical replicates. (**C**) Immunofluorescence on day 15 showing expression of motor neuron progenitor markers Nestin and Olig2, with no GFAP expression. $N = 3$ biological replicates, minimum of three technical replicates. (**D**) Immunofluorescence on day 30 comparing the number of DAPI/Islet 1/2 double-positive cells. Statistical analysis: unpaired *t*-test, no significant difference observed ($p > 0.05$). Mean ± SEM; $N = 3$ biological replicates, three technical replicates of each biological replicate.

© The Author(s)
